# MRI 2-D Parameters Associated with Native Tissue or Transvaginal Mesh Surgical Apical Prolapse Repair Failure

**DOI:** 10.1007/s00192-026-06699-0

**Published:** 2026-05-30

**Authors:** Mark E. Lockhart, Pamela A. Moalli, Holly E. Richter, Isuzu Meyer, Allison C. Weidner, Yuko M. Komesu, Benjamin Carper, Marie G. Gantz

**Affiliations:** 1 Department of Radiology, University of Alabama at Birmingham, Birmingham, AL USA; 2 Department of Obstetrics, Gynecology, and Reproductive Sciences, Division of Urogynecology and Reconstructive Pelvic Surgery, University of Pittsburgh, Pittsburgh, PA USA; 3 Department of Obstetrics and Gynecology, University of Alabama at Birmingham, 619 19th Street South, Birmingham, AL 35243 USA; 4 Department of Obstetrics and Gynecology, Duke University School of Medicine, Durham, NC USA; 5 Department of Obstetrics and Gynecology, University of New Mexico, Albuquerque, NM USA; 6 Social, Statistical, and Environmental Sciences, RTI International, Research Triangle Park, NC USA

**Keywords:** 2-D MRI, Native tissue vaginal hysterectomy, Pelvic organ prolapse, Prolapse surgery, Transvaginal mesh hysteropexy

## Abstract

**Introduction and Hypothesis:**

Magnetic resonance imaging (MRI) can evaluate individual components of postsurgical recurrent prolapse that may not be visible on physical examination and provide insights into mechanisms of prolapse recurrence.

**Methods:**

This planned prospective secondary imaging analysis at nine clinical sites performed in the Study of Uterine Prolapse Procedures-Randomized (SUPeR) trial of the National Institute of Child Health & Human Development (NICHD) Pelvic Floor Disorders Network (ClinicalTrials.gov: NCT01802281) randomized 183 women to native tissue repair (NTR-hysterectomy) or vaginal mesh repair (VM-hysteropexy) for uterovaginal prolapse, and assessed for surgical failure. A subset of participants who enrolled in Defining Mechanisms of Anterior Vaginal Wall Descent (DEMAND) underwent MRI at rest, maximal strain, and post-strain rest (recovery) 30–42 months after surgery or prior to 30 months if undergoing reoperation for recurrence.

**Results:**

Of the 88 participants analyzed (aged 65 ± 7.7 years), 22 (25.0%) had surgical failure by clinical criteria, 8/45 (17.8%) VM-hysteropexy; 14/43 (32.6%) NTR-hysterectomy, *p* = 0.14. Larger genital hiatus (H-line, 86.5 mm vs 72.3 mm, *p* = 0.003), low posterior position during strain (M-line, 55.6 mm vs 40.3 mm, *p* = 0.005), and apical compartments (vaginal apex position below pubococcygeal line (PCL), 42.1 mm vs 13.1 mm, *p* < 0.001) were associated with failure vs success after VM-hysteropexy. In the NTR-hysterectomy group, failures demonstrated greater vaginal width (46.3 mm vs 41.3 mm, *p* = 0.04), vaginal circumference (108.5 mm vs 96.1 mm, *p* = 0.03), and bladder descent vs successes.

**Conclusions:**

Pelvic MRI parameters associated with surgical failure vs success varied between VM-hysteropexy and vaginal hysterectomy.

## Introduction

Pelvic organ prolapse (POP) is a debilitating condition for women, often leading to surgical intervention. Native tissue repairs have higher failure rates, prompting surgeons to use mesh products to provide a more durable repair [1]. The mechanism of failure of either native tissue or mesh-augmented repair is not fully understood, and there was the consideration that 2-D MRI (two dimensional magnetic resonance imaging) evaluation of the impact of the repair on the pelvic compartments may define variables associated with recurrence that may not be visible on physical examination and improve upon rates of surgical failure.

A 2019 Federal Drug Administration (FDA) action removed transvaginal mesh devices from use in the US, but there is still widespread use of transvaginal mesh in Europe and other parts of the world. Two common transvaginal techniques for uterovaginal prolapse exist: vaginal hysterectomy with uterosacral ligament suspension native tissue repair (NTR-hysterectomy) or sacrospinous hysteropexy with vaginal mesh graft (VM-hysteropexy) [2]. Clinical examination of recurrent pelvic organ prolapse uses the Pelvic Organ Quantification (POP-Q) system [3], but while this system can characterize the physical findings on pelvic examination, MRI can provide greater anatomic detail of the pelvic organs and direct visualization of pelvic organ descent in postoperative patients [4].

The objective of this study was to characterize 2-D MRI parameters and evaluate differences in surgical success versus failure following these two apical prolapse repair surgeries to provide insight into mechanisms of failure and how this may vary according to the type of surgery performed. To do this, a priori defined 2-D MRI parameters used in previous studies to compare women with and without prolapse in clinically defined success versus failure cohorts were evaluated.

## Materials and Methods

### Study Design

The Defining Mechanisms of Anterior Vaginal Wall Descent (DEMAND) study is a multi-institutional (nine sites) MRI study that evaluated a subset of women with symptomatic uterovaginal prolapse in the Study of Uterine Prolapse Procedures-Randomized (SUPeR) trial (ClinicalTrials.gov: NCT01802281) [5]. The study was performed with written informed consent and IRB approval from each participating site, and was HIPAA-compliant. The patient population in this report is a subset of the SUPeR trial patient population. Primary outcome data have been previously reported [6], but no two-dimensional MRI results have been published.

### Study Population

From June 2014 to May 2018, participants in the SUPeR trial, after surgical repair of uterovaginal prolapse, were recruited to undergo pelvic MRI at 30–42 months after surgical repair or prior to 30 months if planning to undergo reoperation for recurrent prolapse. There were two groups of participants based upon the surgical procedure: sacrospinous hysteropexy with vaginal mesh (i.e., VM-hysteropexy) and vaginal hysterectomy with uterosacral ligament suspension (i.e., NTR-hysterectomy). Inclusion and exclusion criteria of the DEMAND participants were based upon the SUPeR study, with additional exclusion for MRI contraindications [5].

### MRI Protocol

A standardized static and ultrafast dynamic MRI protocol was created on the basis of existing MRI techniques. MRI parameters for static T2 FSE included TR 2500 ms, TE 102 ms, field of view 20 × 20 cm^2^, slice thickness/gap 3 mm/0 mm, flip angle 180 degrees, and matrix 256 × 256. Parameters for dynamic sagittal ultrafast T2 HASTE included TR 900 ms, TE 80 ms, field of view 36 × 36 cm^2^, slice thickness/gap 4 mm/1 mm, flip angle 90 degrees, refocusing angle 120 degrees, and matrix 320 × 178. All sites trained using the MRI protocol before any study participants were imaged [6]. Participants were also trained on proper strain technique prior to undergoing a dynamic MRI on a 3-Tesla magnetic field (3-T) scanner using a pelvic phased array coil.

All sites trained using the MRI protocol before any study participants were imaged [6]. Participants were also trained on proper strain technique prior to undergoing a dynamic MRI on a 3-Tesla magnetic field (3-T) scanner using a pelvic phased array coil. Prolapse was reduced (as needed) using an MRI-compatible speculum with 10–15 cc of gel in the supine position on the scanner table. Baseline axial, coronal and sagittal T2-weighted pelvic scans were obtained. Sagittal baseline ultrafast images were obtained at rest then repeated during maximal strain for approximately 15 s. At least three strain sequences were imaged with 30 s rest between each. Participants were queried and if poor strain was reported, additional strain sequences were allowed. Lastly, axial post-strain recovery (i.e., at rest with the prolapse non-reduced) T2-weighted images were obtained using the baseline parameters. An MRI quality checklist was completed to ensure study quality and completion, and repeated images were obtained if needed.

### MRI Analysis

MRI images were imported into Osirix Digital Imaging and Communications in Medicine (DICOM) viewer platform for image analysis; no further clinical information was provided. Completely anonymized MRI studies were evaluated by a radiologist with specified measurements recorded on a predetermined data collection form, prior to transfer of the data to the Data Coordinating Center.

Static and dynamic pelvic MRI parameters to assess all three pelvic compartments (anterior, apical, posterior) were selected via literature review prior to any study participant imaging. Published thresholds were applied for normal versus abnormal values based on surgery-naïve cohorts, as follows. Cystocele was diagnosed if bladder neck was 1 cm below pubococcygeal line (PCL) [4]. Urethrocele was diagnosed when the urethra was > 1 cm caudal to the pubic bone. Urethral hypermobility was defined as rotation > 30 degrees from the patient long axis [7, 8]. The angle from patient long axis during strain was compared to the angle from the long axis at rest to determine rotational change. The H-line extended from the posterior–inferior pubic bone to the circular fibers of the posterior rectal wall at the anorectal junction. M-line extended from the posterior circular fibers to a point where the line perpendicularly intersected the PCL. Upper limits for H-line and M-line were defined as 6 cm and 2 cm, respectively [9, 10]. Abnormal cervical descent was defined if it was below the PCL [11, 12]. Enterocele was diagnosed if small bowel projected between the vagina and rectum or into the paravaginal space. Rectocele was defined as anterior rectal wall bulge > 2 cm [13]. For the angle of the posterior plate (the dorsal boundary of the levator plate formed by the iliococcygeal raphe) relative to the PCL, laxity was diagnosed as > 15–20 degrees [8].

Evaluation of levator muscle thickness and whether there were any tears or vaginal asymmetry was also performed [14]. Vaginal asymmetry was diagnosed if the vagina did not have a symmetric H-shape, U-shape (define these), or butterfly-shape configuration. Width of the vagina was measured at the level of greatest transverse diameter, excluding the cranial cervical/vault region. The vaginal circumference was measured at the same level of greatest width.

### Clinical Outcomes

MRI parameters were compared to the reference standard of clinically defined composite surgical success or failure (prolapse recurrence) defined in the SUPeR trial [5]. Surgical failure was based on clinical evaluations during the SUPeR study visit closest to the date of the MRI and was defined as any of the following: report of bothersome vaginal bulge symptoms, prolapse beyond the hymen during the POP-Q exam, or retreatment for prolapse with pessary or surgery. Success was defined as the composite lack of (1) retreatment for prolapse, including pessary or surgery (2) anatomic outcome of any POP-Q measure beyond the hymen, or (3) symptomatic outcomes based upon the Pelvic Floor Distress Inventory question “Do you have a bulge or something falling out that you can see or feel in the vaginal area?” [5].

### Statistical Analysis

Data were analyzed with SAS statistical software (version 9.4, Cary, NC). Baseline demographics and clinical characteristics were compared between surgical repair groups. Linear and logistic models were constructed to compare the participant MRI parameters. Differences between the two repair groups was studied in the context of the model which included surgical group assignment and surgical success/failure as fixed effects, as well as any interaction. For dynamic MRI parameters, the difference between strain and baseline was evaluated by calculating the difference between strain and rest within each individual and then fitting similar linear models as those fit to strain and rest alone. Statistical significance was determined using an alpha of 0.05 and no adjustments were made for multiple testing. Data generated by the authors or analyzed during the study will be made available through the NICHD Data and Specimen Hub (DASH). On the basis of a preliminary inter- and intraobserver reliability study, 40 participants in each surgical repair group were estimated to be necessary for approximately 80% power to detect a moderate difference between the two treatments, but formal sample size calculations were not performed for this primarily descriptive study [6].

## Results

**Fig. 1:**
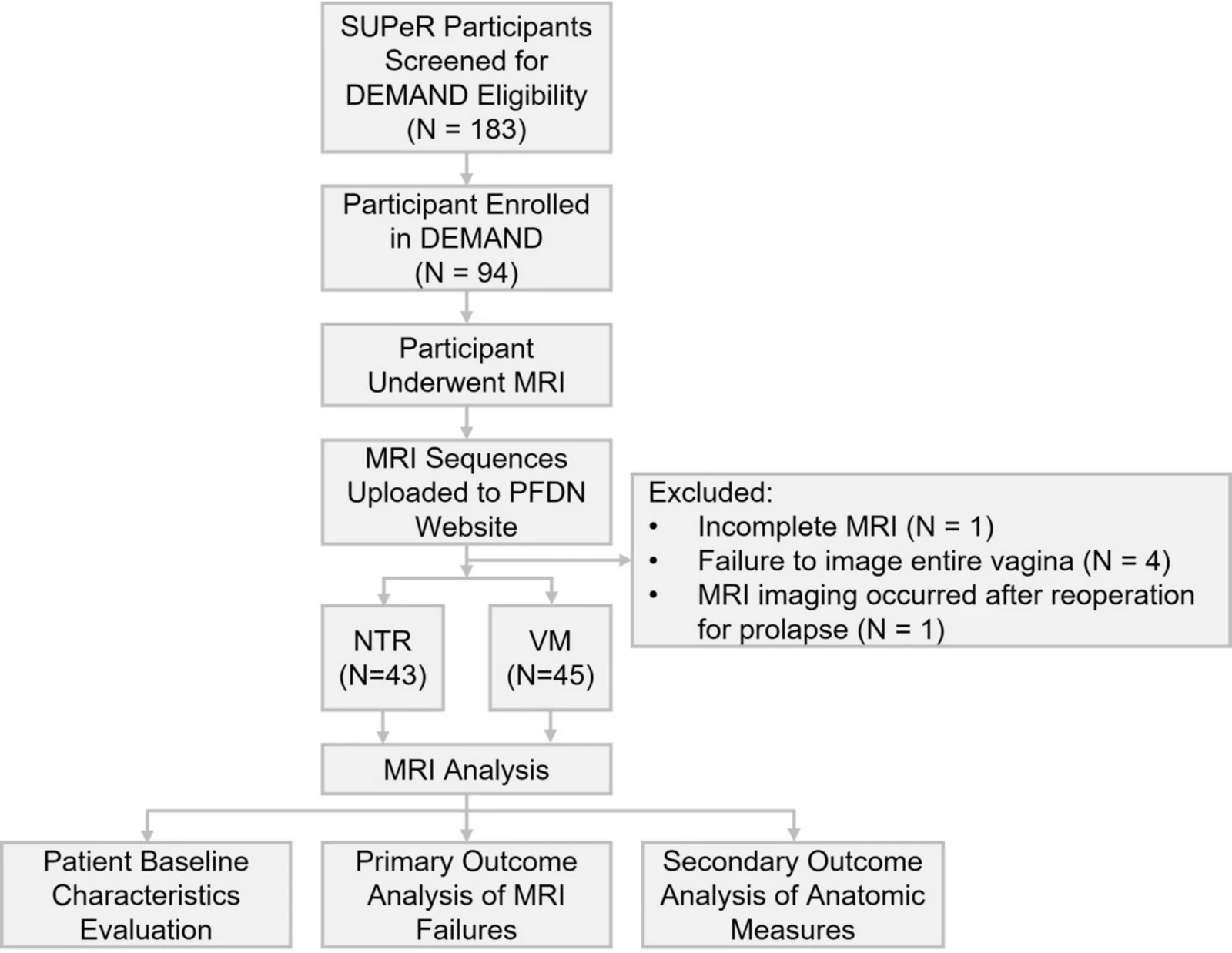
Flow of the Defining Mechanisms of Anterior Vaginal Wall Descent (DEMAND) study

Of the 94 women enrolled in the original DEMAND study, 93 underwent MRI evaluation (Fig. 1). Four participants were excluded from the analysis due to failure to image the entire vagina and one due to failure to complete the examination. Of the remaining 88 participants with evaluable MRI examinations, 45 were in the VM-hysteropexy group and 43 were in the NTR-hysterectomy group. Baseline demographic and clinical characteristics for the VM-hysteropexy and NTR-hysterectomy groups were similar and have been previously published as part of the DEMAND primary results [15]. Briefly, there were no statistically significant differences between the two groups regarding age (years), median (P25, P75), VM-hysteropexy, 63.4 (59.7, 67.8) NTR-hysterectomy 65.4 (60.7, 72.3) *p* = 0.39; race (white), n/N (%), VM-hysteropexy, 36/45 (80), NTR-hysterectomy 36/43 (84), *p* = 0.78; BMI (kg/m^2^), median (P25, P75), VM-hysteropexy, 28.3 (25.4, 31.6), NTR-hysterectomy, 26.4 (24.2, 30.1), *p* = 0.21, or any other characteristics, including gravidity, cesarean section rate, number of vaginal deliveries, POP-Q measurements, or reported outcomes of 11 relevant validated questionnaires related to pelvic symptoms [15].

On the basis of the clinically defined composite surgical outcome, 22/88 (25%) women were identified as having surgical failure, with 8/45 (17.8%) in the VM-hysteropexy group and 14/43 (32.6%) in the NTR-hysterectomy group. The most common site of anatomic failure was the anterior wall of the vagina.

The MRI measures for VM-hysteropexy participants are presented in Table 1. Comparisons between surgical failure and success groups demonstrated that abnormal urethral rotation during strain was well beyond normal surgery-naïve threshold (≤ 30 degrees) in both groups (71.4 and 59.4 degrees, respectively), with no inter-group difference (*p* = 0.42**)**.

**Table 1:** MRI outcomes for mesh (VM-hysteropexy) participants based on clinical outcome at 36 months

Parameter	Setting	Clinical failure (*N* = 8), mean (SE) or *N* (%)	Clinical success (*N* = 37), mean (SE) or *N* (%)	Model-estimated mean difference or odds ratio [95% CI]	*P* value of comparison^a^
Dynamic MRI parameters					
H-line (mm)^b^	Rest	68.0 (2.8)	63.8 (1.3)	4.2 [−1.9, 10.3]	0.18
	Strain	86.5 (4.2)	72.3 (2.0)	14.3 [5.0, 23.6] *	**0.003**
	Difference (rest- strain)^b^	18.6 (3.7)	8.5 (1.7)	10.1 [1.9, 18.2] *	**0.02**
M-line (mm)^b^	Rest	34.0 (2.8)	28.2 (1.3)	5.8 [−0.4, 12.0]	0.07
	Strain	55.6 (4.8)	40.3 (2.3)	15.3 [4.7, 25.9] *	**0.005**
	Difference (rest- strain)^b^	21.6 (4.5)	12.1 (2.1)	9.5 [−0.4, 19.4]	0.06
BN-line (mm)^d^	Rest	1.5 (4.5)	6.0 (2.1)	−4.4 [−14.2, 5.4]	0.37
	Strain	−27.2 (6.2)	−19.3 (2.9)	−7.9 [−21.4, 5.7]	0.25
	Difference (rest- strain)^d^	−28.7 (5.3)	−25.3 (2.5)	−3.4 [−15.1, 8.3]	0.56
Vaginal length (mm)^b^	Rest	61.7 (5.0)	61.3 (2.3)	0.4 [−10.5, 11.4]	0.94
	Strain	46.5 (6.0)	56.1 (2.8)	−9.6 [−22.8, 3.6]	0.15
	Difference (rest- strain)^b^	−15.2 (5.8)	−5.2 (2.7)	−10.0 [−22.7, 2.7]	0.12
Cervix distance from PCL (mm)^d^	Rest	−1.0 (4.9)	10.1 (2.3)	−11.1 [−21.9, −0.3] *	**0.04**
	Strain	−42.1 (6.1)	−13.1 (2.8)	−29.0 [−42.4, −15.6] *	** < 0.001**
	Difference (rest- strain)^d^	−41.1 (5.4)	−23.2 (2.5)	−17.9 [−29.8, −6.0] *	**0.004**
Posterior plate angle (degrees)^d^	Rest	−18.8 (4.5)	−16.0 (2.1)	−2.7 [−12.6, 7.1]	0.58
	Strain	−36.5 (5.9)	−30.9 (2.8)	−5.6 [−18.6, 7.4]	0.39
	Difference (rest- strain)^d^	−17.8 (5.0)	−14.9 (2.3)	−3.1 [−13.9, 8.1]	0.60
Urethral angle (degrees)^c^	Rest	−2.5 (9.4)	−5.6 (4.4)	−2.0 [−19.2, 15.2]	0.77
	Strain	−71.4 (13.5)	−59.4 (6.3)	−23.8 [−47.8, 0.2]	0.42
	Difference (rest- strain)^c^	−68.9 (12.1)	−53.8 (5.6)	−21.8 [−43.8, 0.2]	0.26
Presence of urethrocele	Rest	0 (0.0)	3 (8.1)	0.00 [0.00, –]	0.98
	Strain	6 (75.0)	20 (54.1)	3.33 [0.75, 14.73]	0.29
	Change	6 (75.0)	17 (50.0)	4.10 [0.91, 18.55]	0.22
Static MRI parameter					
Levator hiatal circumference (mm)^b^	Rest	275.3 (12.8)	253.0 (6.0)	22.4 [−5.7, 50.5]	0.12
	Recovery	278.7 (12.4)	261.8 (5.7)	16.9 [−10.2, 44.0]	0.22
	Difference (rest-recovery)	3.4 (3.9)	8.8 (1.8)	−5.4 [−14.0, 3.1]	0.21
Levator hiatal width (mm)^b^	Rest	76.6 (4.5)	71.4 (2.1)	5.2 [−4.8, 15.1]	0.30
	Recovery	78.9 (4.7)	73.5 (2.2)	5.5 [−4.7, 15.7]	0.29
	Difference (rest-recovery)	2.4 (1.3)	2.1 (0.6)	0.3 [−2.6, 3.2]	0.84
Levator hiatal area (cm^2^)^b^	Rest	56.3 (5.5)	46.9 (2.5)	9.4 [−2.6, 21.4]	0.12
	Recovery	58.4 (5.4)	50.1 (2.5)	8.2 [−3.6, 20.1]	0.17
	Difference (rest-recovery)	2.1 (1.6)	3.2 (0.7)	−1.2 [−4.6, 2.2]	0.50
Vaginal width (mm)	Rest	49.7 (2.6)	44.4 (1.2)	5.3 [−0.4, 11.0]	0.07
Vaginal circumference (mm)	Rest	111.0 (6.2)	101.9 (2.9)	9.0 [−4.7, 22.8]	0.19
Pubococcygeal line (PCL) (mm)		108.7 (3.0)	105.8 (1.4)	3.0 [−3.6, 9.5]	0.37
Presence of rectocele		3 (37.5)	17 (45.9)	0.71 [0.14, 3.47]	0.66
Presence of enterocele		0 (0.0)	0 (0.0)	1.00 [0.00, –]	1.00
Presence of paravaginal defect		2 (25.0)	4 (10.8)	2.75 [0.40, 19.04]	0.30
Presence of vaginal asymmetry		1 (12.5)	1 (2.7)	5.14 [0.27, 96.30]	0.27

^a^*P* values are based on a linear model for continuous outcomes and logistic model for binary outcomes. All tests were conducted at a 0.05 significance level

^b^Increase in value (length or area) is given by a positive value; decrease in length is given by a negative value

^c^Anterior movement is given by a positive value; posterior movement is given by a negative value

^d^Superior movement is given by a positive value; inferior movement is given by a negative value

In VM-hysteropexy participants, the H-line (anterior–posterior [AP] diameter of genital hiatus) and M-line (vertical position of posterior margin of the hiatus) in both failures and successes were beyond the surgically naïve normal thresholds of 6 cm and 2 cm, respectively. The H-line during strain was larger in the prolapse failures than successes (86.5 vs 72.3 mm, *p* = 0.003), and there was greater change/expansion of the genital hiatus at strain relative to at rest for failures compared to successes (18.6 vs 8.5 mm change, *p* = 0.02). Posterior compartment descent during strain, as represented by the M-line, was greater in failures than successes (55.6 vs 40.3 mm, *p* = 0.005). Greater posterior plate descent below the PCL occurred in both surgical failure and success groups (posterior plate angle 36.5 and 30.9 degrees, respectively; normal < 20 degrees), and did not differ between groups. The change in posterior plate at strain relative to baseline in failures versus successes (17.8 vs 14.9 degrees) was not significantly different.

**Fig. 2:**
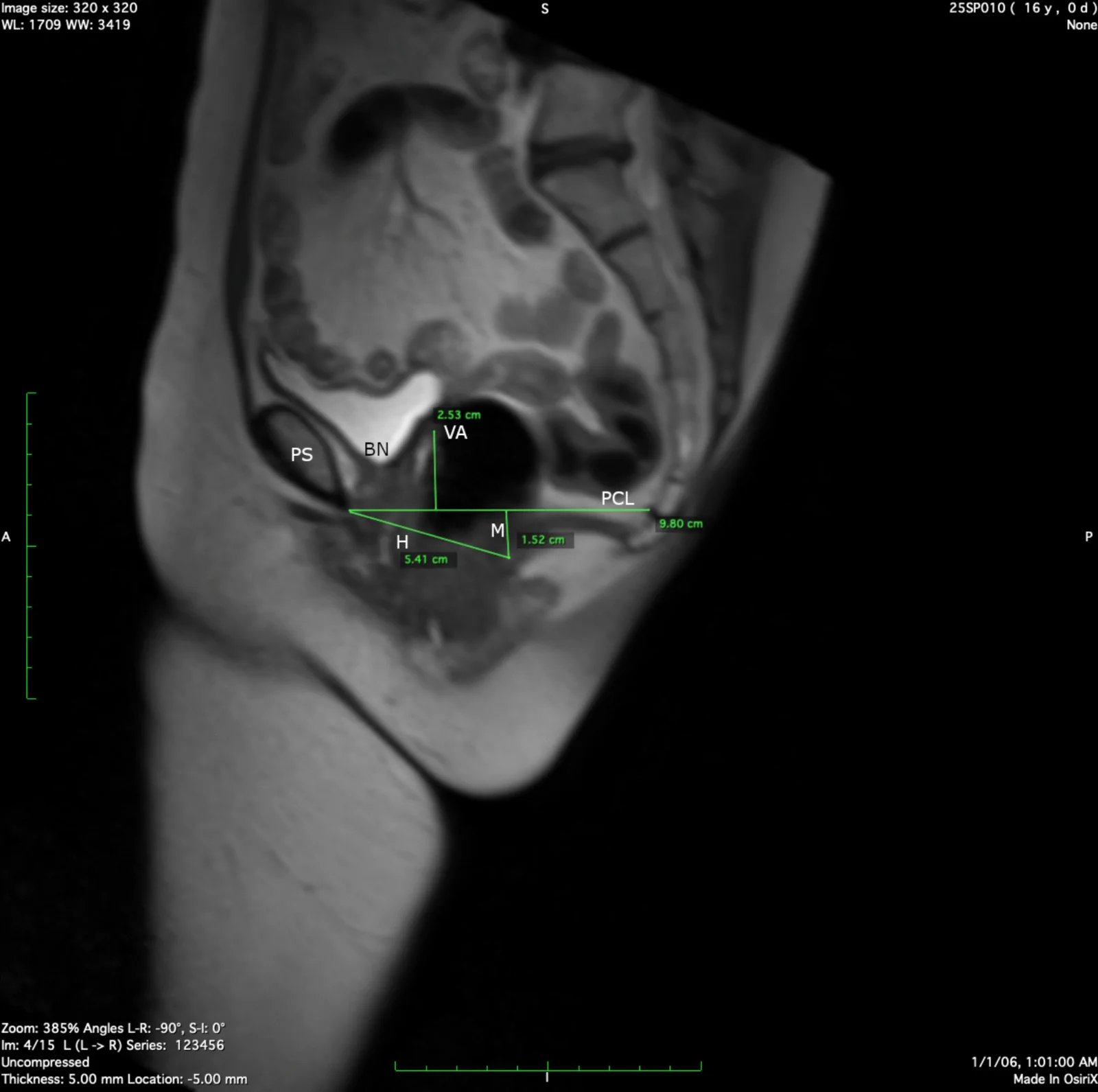

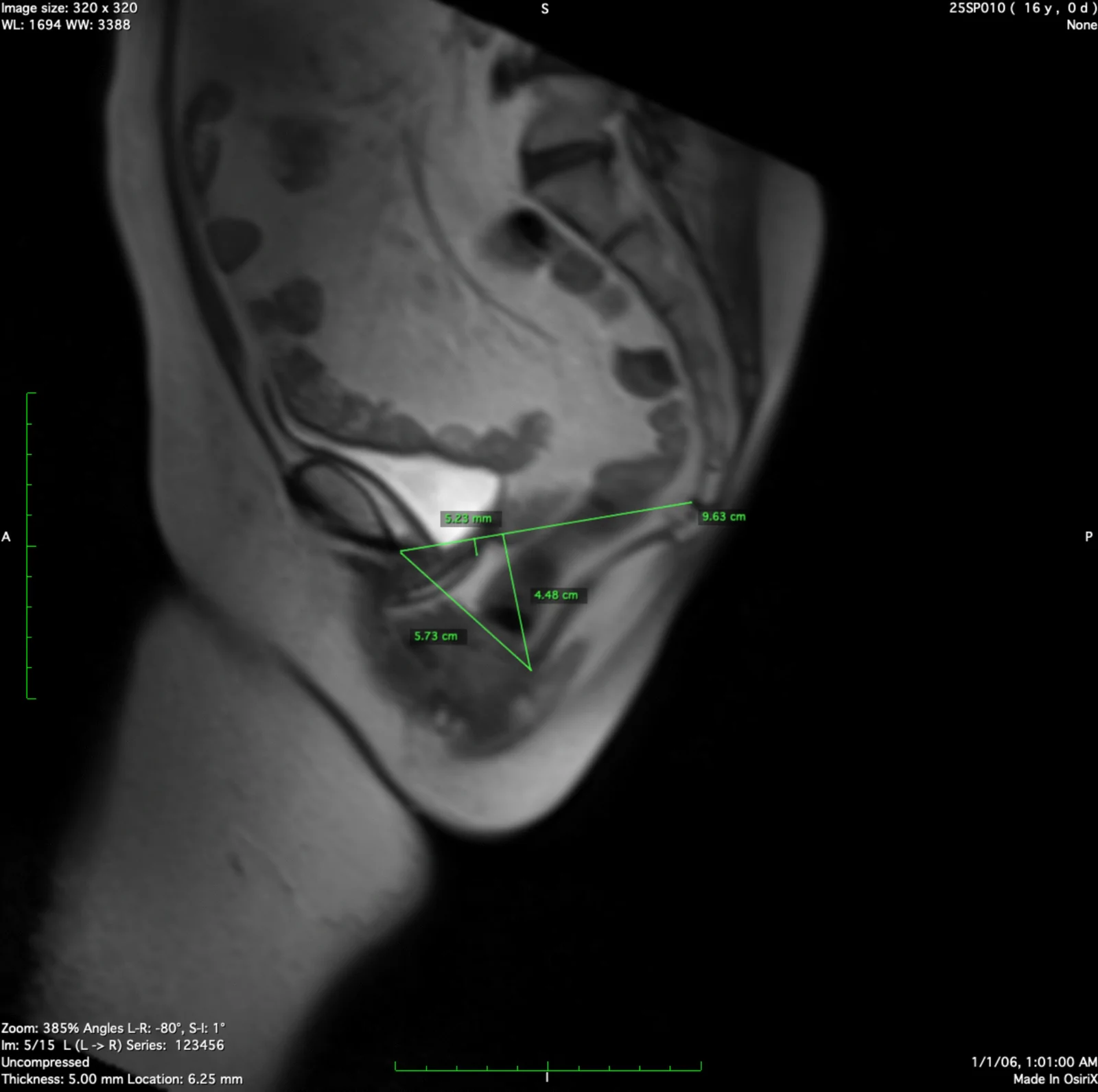
Pelvic organ descent in VM-hysteropexy participant with clinically unsuccessful repair. **a**) Sagittal image at rest shows normal pelvic organ position of bladder neck above PCL, vaginal apex 2.53 cm above PCL, H-line 5.41 cm, and M-line 1.52 cm, and normal posterior plate angle. **b**) Sagittal image during strain shows normal pelvic organ measurements of bladder neck at the PCL, vaginal apex 0.52 cm below PCL, and H-line 5.73 cm. Although the apical compartment is near the PCL, it is markedly lower than at baseline. The M-line 4.48 cm and posterior plate angle are abnormal and increased from baseline. PCL, pubococcygeal line. PS, pubic symphysis. BN, bladder neck. VA, vaginal apex. M, M-line. H, H-line

Apical prolapse in VM-hysteropexy participants was more common in surgical failures than successes (Fig. 2, A/B). The cervix demonstrated greater descent position during strain in failures vs successes (42.1 vs 13.1 mm below PCL, respectively, *p* < 0.001) and greater cervical descent from baseline rest images (41.1 vs 23.2 mm, respectively, *p* = 0.004).

Rectoceles were similarly common in VM-hysteropexy surgical failures and successes (3/8 (37.5%) and 17/37 (45.9%), respectively *p* = 0.66). Although paravaginal defects, levator tears, and vaginal asymmetry were more frequent in VM-hysteropexy recurrences, the number was too small for statistical comparison. No enteroceles were identified. There was no difference in AP pelvimetry measurements for surgical failures versus successes.

**Fig. 3:**
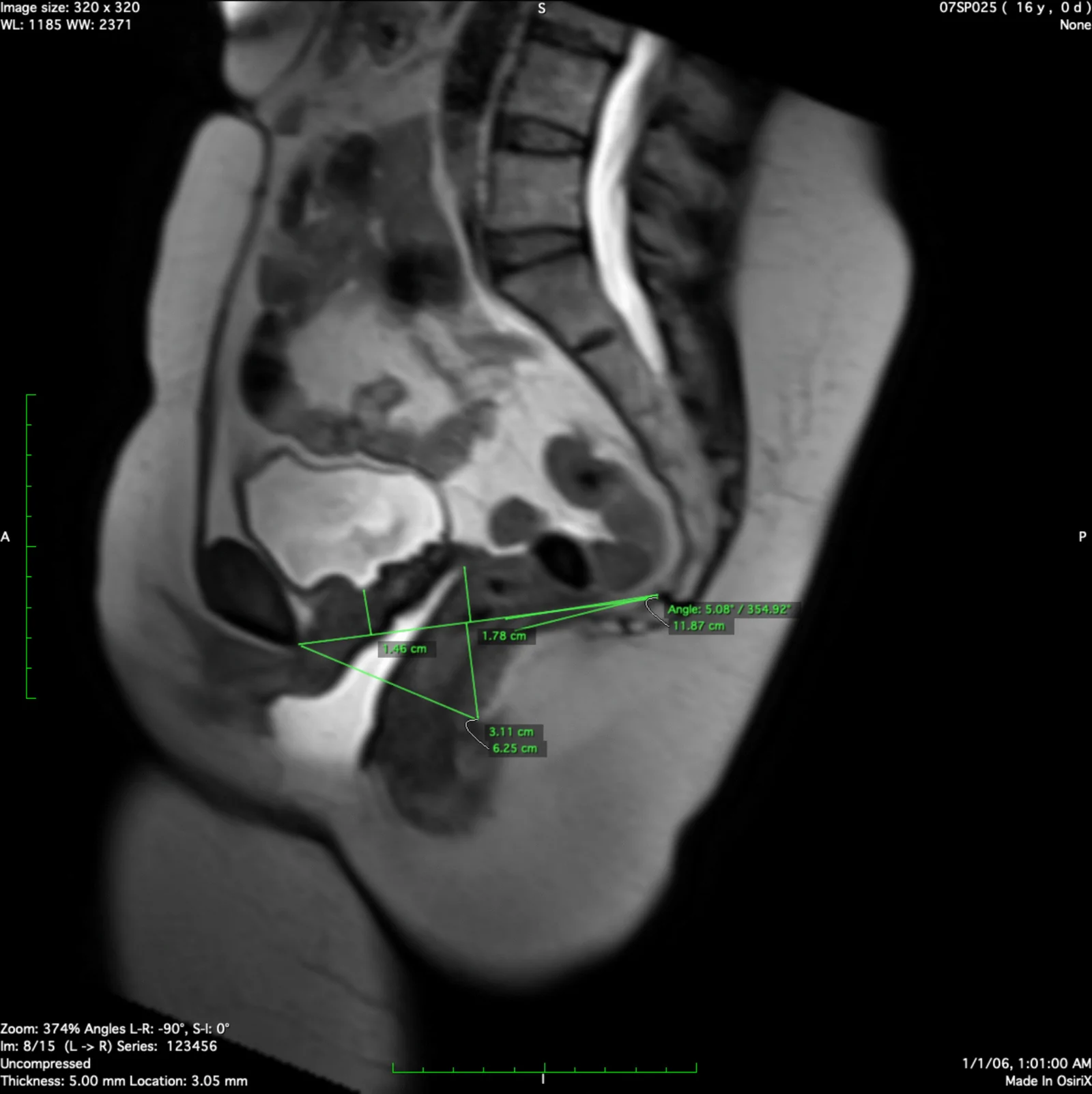

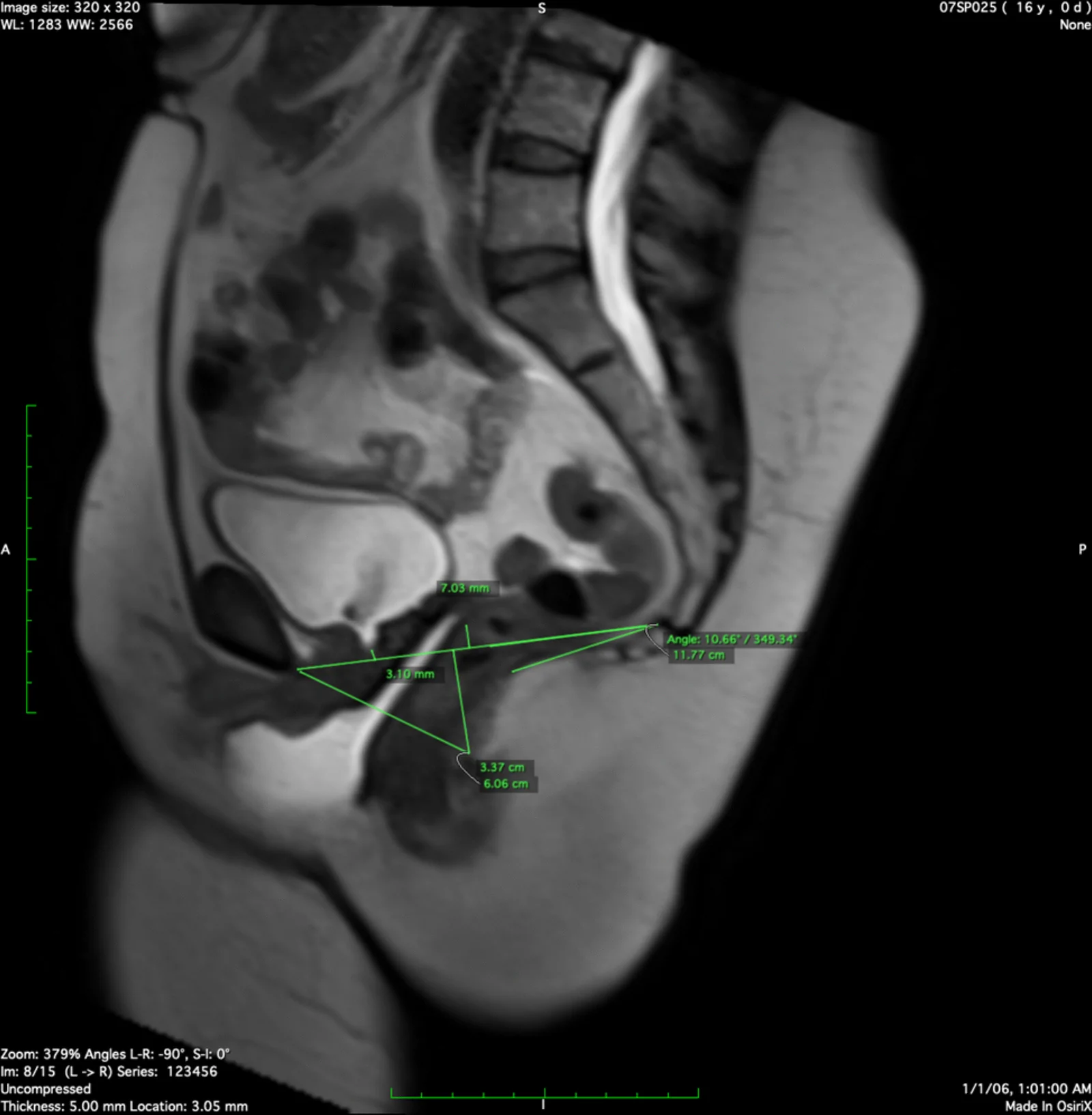
Mild pelvic floor descent in NTR-hysterectomy participant with clinically unsuccessful repair. (**a**) Sagittal image at rest shows normal pelvic organ measurements of bladder neck 1.46 cm above PCL, vaginal apex 1.78 cm above PCL, and posterior plate angle 5 degrees. Distances of the H-line 6.25 cm and M-line 3.11 cm are abnormal. (**b**) Sagittal image during strain shows normal pelvic organ measurements of bladder neck 0.3 cm above PCL, vaginal apex 0.73 cm above PCL, and posterior plate angle 10.6 degrees. Distances of the H-line 6.07 cm and M-line 3.37 cm are abnormal but not worsened from baseline. PCL, pubococcygeal line

The MRI measures for NTR-hysterectomy participants are shown in Table 2. Comparisons between surgical failures and successes demonstrated lower position of the bladder neck at baseline (4.3 mm below PCL vs 8.4 mm above PCL, *p* = 0.003), and lower position during strain (40.6 mm below PCL vs 26.0 mm below PCL, *p* = 0.01) in failures relative to successes. There were no other dynamic differences found. Urethrocele during strain occurred in both failures and successes (10/14 (71.4%) and 14/29 (48.3%), *p* = 0.16). The H-line (AP diameter of genital hiatus) and M-line (vertical position of posterior hiatus margin) in both failures and successes were beyond the normal values of 6 cm and 2 cm, respectively. The H-line during strain was similar in the prolapse failures and successes (75.8 vs 73.4 mm, *p* = 0.54), and there was similar change/expansion of the genital hiatus at strain relative to at rest for failures compared to successes (10.0 vs 9.7 mm change, *p* = 0.93) (Fig. 3 a/b).

**Table 2:** MRI outcomes for NTR-hysterectomy participants based on clinical outcome at 36 months

Parameter	Setting	Clinical failure (*N* = 14), mean (SE) or *N* (%)	Clinical success (*N* = 29), mean (SE) or *N* (%)	Model-estimated mean difference or odds Ratio [95% CI]	*P* value of comparison^a^
Dynamic MRI parameter (mean, SD)					
H-line (mm)^b^	Rest	65.8 (2.1)	63.7 (1.5)	2.1 [−3.0, 7.2]	0.41
	Strain	75.8 (3.2)	73.4 (2.2)	2.4 [−5.3, 10.2]	0.54
	Difference (rest- strain)^b^	10.0 (2.8)	9.7 (2.0)	0.3 [−6.5, 7.1]	0.93
M-line (mm)^b^	Rest	30.5 (2.1)	27.3 (1.5)	3.2 [−2.0, 8.4]	0.22
	Strain	43.1 (3.7)	40.6 (2.5)	2.4 [−6.4, 11.3]	0.59
	Difference (rest- strain)^b^	12.6 (3.4)	13.4 (2.4)	−0.8 [−9.0, 7.5]	0.85
BN-line (mm)^d^	Rest	−4.3 (3.4)	8.4 (2.3)	−12.6 [−20.8, −4.5] *	**0.003**
	Strain	−40.6 (4.7)	−26.0 (3.3)	−14.6 [−25.9, −3.2] *	**0.01**
	Difference (rest- strain)^d^	−36.3 (4.0)	−34.4 (2.8)	−1.9 [−11.7, 7.9]	0.70
Vaginal length (mm)^b^	Rest	54.6 (3.8)	59.4 (2.6)	−4.9 [−14.0, 4.3]	0.29
	Strain	50.9 (4.5)	49.8 (3.2)	1.0 [−10.0, 12.0]	0.85
	Difference (rest- strain)^b^	−3.7 (4.4)	−9.6 (3.0)	5.9 [−4.7, 16.5]	0.27
Cervix distance from PCL (mm)^d^	Rest	13.5 (3.7)	19.7 (2.6)	−6.2 [−15.2, 2.8]	0.18
	Strain	−14.1 (4.6)	−5.6 (3.2)	−8.5 [−19.7, 2.6]	0.13
	Difference (rest- strain)^d^	−27.7 (4.1)	−25.3 (2.8)	−2.4 [−12.3, 7.6]	0.64
Posterior plate angle (degrees)^d^	Rest	−17.8 (3.5)	−13.2 (2.4)	−4.6 [−13.1, 3.9]	0.28
	Strain	−27.4 (4.6)	−28.5 (3.2)	1.1 [−10.1, 12.3]	0.85
	Difference (rest- strain)^d^	−9.5 (3.9)	−15.2 (2.7)	5.7 [−3.8, 15.1]	0.24
Urethral angle (degrees)^c^	Rest	−22.6 (7.4)	−6.4 (5.0)	−16.2 [−33.9, 1.6]	0.07
	Strain	−84.5 (10.6)	−71.3 (7.2)	−13.2 [−38.7, 12.3]	0.31
	Difference (rest- strain)^c^	−61.8 (9.5)	−64.9 (6.5)	3.0 [−19.8, 25.8]	0.79
Presence of urethrocele	Rest	5 (35.7)	4 (13.8)	3.47 [0.74, 16.23]	0.11
	Strain	10 (71.4)	14 (48.3)	2.68 [0.67, 10.75]	0.16
	Change	5 (55.6)	10 (40.0)	1.87 [0.39, 8.97]	0.43
Static MRI parameter (mean, SD)					
Levator hiatus circumference (mm)^b^	Rest	265.8 (9.7)	253.0 (6.7)	12.8 [−10.7, 36.2]	0.28
	Recovery	271.1 (9.3)	257.1 (6.5)	13.9 [−8.7, 36.6]	0.22
	Difference (rest-recovery)	5.2 (2.9)	4.1 (2.0)	1.2 [−6.0, 8.3]	0.75
Levator hiatus width (mm)^b^	Rest	75.8 (3.4)	67.8 (2.4)	7.9 [−0.4, 16.2]	0.06
	Recovery	77.3 (3.5)	70.2 (2.4)	7.1 [−1.4, 15.7]	0.10
	Difference (rest-recovery)	1.5 (1.0)	2.3 (0.7)	−0.8 [−3.2, 1.7]	0.53
Levator hiatus area (cm^2^)^b^	Rest	53.7 (4.1)	46.2 (2.9)	7.5 [−2.5, 17.5]	0.14
	Recovery	55.1 (4.1)	47.9 (2.8)	7.2 [−2.7, 17.1]	0.15
	Difference (rest-recovery)	1.4 (1.2)	1.7 (0.8)	−0.3 [−3.2, 2.5]	0.83
Vaginal width at rest (mm)	Rest	46.3 (1.9)	41.3 (1.4)	5.0 [0.3, 9.7] *	**0.04**
Vaginal circumference at rest (mm)	Rest	108.5 (4.7)	96.1 (3.3)	12.4 [1.0, 23.9] *	**0.03**
Pubococcygeal line (PCL) (mm)		106.9 (2.2)	104.6 (1.6)	2.2 [−3.2, 7.7]	0.42
Presence of rectocele		5 (35.7)	14 (50.0)	0.56 [0.15, 2.12]	0.39
Presence of enterocele		1 (7.1)	0 (0.0)	– [0.00, –]	0.98
Presence of paravaginal defect		6 (42.9)	5 (17.2)	3.60 [0.84, 15.38]	0.08
Presence of vaginal asymmetry		1 (7.1)	4 (13.8)	0.48 [0.05, 4.92]	0.53

^a^*P* values are based on linear models for continuous outcomes and logistic models for binary outcomes. All tests were conducted at a 0.05 significance level

^b^increase in value (length or area) is given by positive value; decrease in length is given by negative value

^c^anterior movement is given by positive value; posterior movement is given by negative value

^d^superior movement is given by positive value; inferior movement is given by negative value

The vaginal width was larger in failures than successes (46.3 mm vs 41.3 mm, *p* = 0.04) as was the vaginal circumference (108.5 mm vs 96.1 mm, *p* = 0.03). The posterior compartment was abnormal during strain in both failures and successes (posterior plate angle 27.4 and 28.5 degrees below PCL; normal < 20 degrees). Abnormal low vaginal apex position meeting existing criteria for apical prolapse was present in failures but not successes (14.1 mm (SD 4.6 mm) vs 5.6 mm (SD 3.2 mm) below PCL); however, this difference did not reach statistical significance, and the distance of descent relative to baseline was not different.

Some parameters such as rectocele and vaginal asymmetry had higher numbers in successes than failures, but the numbers were too small for statistical comparison. There was no difference in pelvimetry or levator muscle grades in surgical successes vs failures.

Overall, differences in parameters between surgical failures versus successes also varied according to the type of surgery performed (Table 3). For certain MRI parameters, the differences in measures between prolapse recurrences and successes were greater in VM-hysteropexy participants than in those with NTR-hysterectomy.

**Table 3:** MRI outcomes for VM-hysteropexy vs NTR-hysterectomy participants based on clinical outcome at 36 months

Parameter	Setting	Model-estimated mean difference or odds ratio [95% CI]		Difference in difference [95% CI]	*P* value of comparison^a^
		**VM-hysteropexy**	**NTR-hysterectomy**		
Dynamic MRI parameter (mean, SD)					
H-line (mm)^b^	Rest	4.2 [−1.9, 10.3]	2.1 [−3.0, 7.2]	2.1 [−5.9, 10.1]	0.60
	Strain	14.3 [5.0, 23.6] *	2.4 [−5.3, 10.2]	11.8 [−0.3, 24.0]	0.05
	Difference (rest- strain)^b^	10.1 [1.9, 18.2] *	0.3 [−6.5, 7.1]	9.8 [−0.9, 20.4]	0.07
M-line (mm)^b^	Rest	5.8 [−0.4, 12.0]	3.2 [−2.0, 8.4]	2.6 [−5.5, 10.7]	0.53
	Strain	15.3 [4.7, 25.9] *	2.4 [−6.4, 11.3]	12.9 [−0.9, 26.7]	0.07
	Difference (rest- strain)^b^	9.5 [−0.4, 19.4]	−0.8 [−9.0, 7.5]	10.3 [−2.6, 23.2]	0.12
BN-line (mm)^d^	Rest	−4.4 [−14.2, 5.4]	−12.6 [−20.8, −4.5] *	8.2 [−4.6, 21.0]	0.20
	Strain	−7.9 [−21.4, 5.7]	−14.6 [−25.9, −3.2] *	6.7 [−11.0, 24.4]	0.45
	Difference (rest- strain)^d^	−3.4 [−15.1, 8.3]	−1.9 [−11.7, 7.9]	−1.5 [−16.7, 13.7]	0.85
Vaginal length (mm)^b^	Rest	0.4 [−10.5, 11.4]	−4.9 [−14.0, 4.3]	5.3 [−8.9, 19.5]	0.46
	Strain	−9.6 [−22.8, 3.6]	1.0 [−10.0, 12.0]	−10.6 [−27.8, 6.6]	0.22
	Difference (rest- strain)^b^	−10.0 [−22.7, 2.7]	5.9 [−4.7, 16.5]	−15.9 [−32.4, 0.6]	0.06
Cervix distance from PCL (mm)^d^	Rest	−11.1 [−21.9, −0.3] *	−6.2 [−15.2, 2.8]	−4.9 [−19.0, 9.2]	0.49
	Strain	−29.0 [−42.4, −15.6] *	−8.5 [−19.7, 2.6]	−20.5 [−37.9, −3.0] *	**0.02**
	Difference (rest- strain)^d^	−17.9 [−29.8, −6.0] *	−2.4 [−12.3, 7.6]	−15.6 [−31.1, −0.1] *	**0.05**
Posterior plate angle (degrees)^d^	Rest	−2.7 [−12.6, 7.1]	−4.6 [−13.1, 3.9]	−1.9 [−14.8, 11.1]	0.77
	Strain	−5.6 [−18.6, 7.4]	1.1 [−10.1, 12.3]	6.7 [−10.4, 23.8]	0.44
	Difference (rest- strain)^d^	−3.1 [−13.9, 8.1]	5.7 [−3.8, 15.1]	8.6 [−5.9, 23.0]	0.24
Urethral angle (degrees)^c^	Rest	3.1 [−17.6, 23.7]	−16.2 [−33.9, 1.6]	19.3 [−8.0, 46.5]	0.16
	Strain	−12.0 [−41.6, 17.7]	−13.2 [−38.7, 12.3]	1.2 [−37.9, 40.3]	0.95
	Difference (rest- strain)^c^	−15.0 [−41.6, 11.5]	3.0 [−19.8, 25.8]	−18.0 [−53.0, 16.9]	0.31
Presence of urethrocele	Rest	0.00 [0.00, I]	3.47 [0.74, 16.23]	0.00 [0.00, I]	0.98
	Strain	2.55 [0.44, 14.69]	2.68 [0.67, 10.75]	0.95 [0.10, 8.90]	0.97
	Change	3.00 [0.51, 17.53]	1.87 [0.39, 8.97]	1.60 [0.15, 16.94]	0.69
Static MRI parameter (mean, SD)					
Levator hiatus circumference (mm)^b^	Rest	22.4 [−5.7, 50.5]	12.8 [−10.7, 36.2]	9.6 [−27.0, 46.2]	0.60
	Recovery	16.9 [−10.2, 44.0]	13.9 [−8.7, 36.6]	3.0 [−32.3, 38.3]	0.87
	Difference (rest-recovery)	−5.4 [−14.0, 3.1]	1.2 [−6.0, 8.3]	−6.6 [−17.7, 4.5]	0.24
Levator hiatus width (mm)^b^	Rest	5.2 [−4.8, 15.1]	7.9 [−0.4, 16.2]	−2.7 [−15.7, 10.2]	0.68
	Recovery	5.5 [−4.7, 15.7]	7.1 [−1.4, 15.7]	−1.7 [−15.0, 11.6]	0.80
	Difference (rest-recovery)	0.3 [−2.6, 3.2]	−0.8 [−3.2, 1.7]	1.1 [−2.7, 4.9]	0.58
Levator hiatus area (cm^2^)^b^	Rest	9.4 [−2.6, 21.4]	7.5 [−2.5, 17.5]	1.9 [−13.7, 17.5]	0.81
	Recovery	8.2 [−3.6, 20.1]	7.2 [−2.7, 17.1]	1.0 [−14.4, 16.5]	0.89
	Difference (rest-recovery)	−1.2 [−4.6, 2.2]	−0.3 [−3.2, 2.5]	−0.9 [−5.3, 3.6]	0.70
Vaginal width at rest (mm)	Rest	5.3 [−0.4, 11.0]	5.0 [0.3, 9.7] *	0.3 [−7.0, 7.7]	0.93
Vaginal circumference at rest (mm)	Rest	9.0 [−4.7, 22.8]	12.4 [1.0, 23.9] *	−3.4 [−21.3, 14.5]	0.71
Pubococcygeal line (PCL) (mm)		3.0 [−3.6, 9.5]	2.2 [−3.2, 7.7]	0.7 [−7.8, 9.2]	0.86
Presence of rectocele		0.71 [0.14, 3.47]	0.56 [0.15, 2.12]	1.27 [0.16, 10.19]	0.82
Presence of enterocele		1.00 [0.00, –]	– [0.00, –]	0.00 [0.00, –]	0.99
Presence of paravaginal defect		2.75 [0.40, 19.04]	3.60 [0.84, 15.38]	0.76 [0.07, 8.59]	0.83
Presence of vaginal asymmetry		5.14 [0.27, 96.30]	0.48 [0.05, 4.92]	10.70 [0.25, 450.44]	0.21

^a^*P* values are based on linear models for continuous outcomes and logistic models for binary outcomes. All tests were conducted at a 0.05 significance level

^b^increase in value (length or area) is given by positive value; decrease in length is given by negative value

^c^anterior movement is given by positive value; posterior movement is given by negative value

^d^superior movement is given by positive value; inferior movement is given by negative value

The apex (point C by POP-Q) measure descended more during strain in failures relative to successes for VM-hysteropexy participants than for NTR-hysterectomy participants (42.1 mm vs 13.1 mm for failure vs success in VM-hysteropexy, and 14.1 mm vs 5.6 mm for failure vs success in NTR-hysterectomy, *p* = 0.02). There was also a greater difference in the change between strain and rest at the apex for surgical failures vs successes in the VM-hysteropexy group compared to the NTR-hysterectomy group (41.1 mm vs 23.2 mm for failure vs success in VM-hysteropexy, and 27.7 mm vs 25.3 mm for failure vs success in NTR-hysterectomy, *p* = 0.05).

## Discussion

In both the sacrospinous hysteropexy with vaginal mesh (i.e., VM-hysteropexy) and vaginal hysterectomy with uterosacral ligament suspension (i.e., NTR-hysterectomy) surgical groups, support of the pelvic structures was altered relative to the surgically naïve state. While it is reasonable to assume that the movement of the pelvic organs may be altered by surgery, this has not been previously completely described by MRI. The primary mechanism of recurrences in the DEMAND population was anterior and apical compartment descent. Cystocele on MRI correlated well with surgical failures but other measures of anterior compartment laxity such as urethrocele or urethral hypermobility were present in participants regardless of failure or success. Greater inferior displacement of the vaginal apex was observed among surgical failures with VM-hysteropexy but less so in failures after NTR-hysterectomy. This may reflect attenuation of support by the mesh arms.

MRI parameters differed between the NTR-hysterectomy and VM-hysteropexy participants in amplitude and location. All groups had at least some sign of persistent anterior compartment descent, noted to be greater in those with surgical failure. The magnitudes of abnormal values in the posterior and apical compartments were more closely associated with surgical failure in VM-hysteropexy participants than those after NTR-hysterectomy. A possible explanation is that more participants in the NTR cohort who currently have surgical success were closer to thresholds that meet the definition of recurrence than those in the mesh group.

There is an increasing amount of data in the literature regarding thresholds on MRI for the diagnosis of POP. The current results suggest that many of these parameters are greater in patients with surgical failures relative to those considered surgical successes. What is unexpected is the common presence of an abnormally positioned anterior compartment in both failures and successes. While these values are greater for failures, a substantial number of patients have significant pelvic organ descent despite lack of symptoms or signs of recurrent prolapse compared to historic prolapse naive patients. It is uncertain whether these patients will be at increased risk for long-term development of symptoms or objective findings.

The expansion of the H-line and the descent represented by the M-line capture the multi-compartmental effects of prolapse recurrence. In the current study, these changes were more prominent after VM-hysteropexy than NTR-hysterectomy possibly due to the differences in suspension points based upon the two techniques.

Strengths of this study include characterization of 2-D MRI characteristics in a very well-phenotyped population of women undergoing prolapse surgery. The MRI technique was standardized, and outcomes were objectively assessed at a single site to minimize variability. The primary limitation of this secondary analysis is that the DEMAND population is a subset of SUPeR patients, and those enrolled in the study may not represent the overall population and, thus, bias cannot be fully excluded. In addition, the extent of prolapse observed may be underestimated since MRI studies were performed in the supine position, which is current standard of care. Strain was performed after patient training and patients were asked to repeat the maneuver if they felt that had not achieved full strain, but lack of defecation could reduce sensitivity. Previously, we reported on this cohort based on a 3-D analysis which also found the anterior compartment was the most common site of failure [15]. There are small differences in the 2-D and 3-D evaluation as the 2-D results were derived from a single plane. Also, this study used a clinical definition of failure, whereas the 3-D results were relative to POP-Q measurements beyond the hymen on the MRIs.

In conclusion, in participants with clinically defined surgical success after transvaginal prolapse repair, based on MRI parameters, there was evidence of multi-compartmental descent compared to thresholds based on measures from surgery-naïve cohorts. There is greater difference in the descent of posterior and apical compartments between successes and failures in participants with VM-hysteropexy than those undergoing NTR-hysterectomy. After NTR-hysterectomy, bladder descent is greater in failures than successes, and a more pronounced difference between failures and successes was present after NTR-hysterectomy compared to VM-hysteropexy. Urethrocele and urethral hypermobility are present in all groups regardless of intervention arm or clinical outcome. In this prospective evaluation of participants with surgical success or failure, many of the existing objective MRI criteria do not closely align with clinically defined outcomes and should be used with caution in the absence of clinical symptoms or objective findings.

## Data Availability

The data for the trial is publicly available through the NICHD Data and Specimen Hub.
